# Coping Mechanism Adopted by Medical Interns to Manage Stress: A Mixed Methods Study

**DOI:** 10.7759/cureus.107439

**Published:** 2026-04-21

**Authors:** Ayesha T Aejaz, Mubeena Haleema

**Affiliations:** 1 Respiratory Medicine, Sagar Hospitals, Bengaluru, IND; 2 Community Medicine, Prathima Institute of Medical Sciences, Karimnagar, IND

**Keywords:** brief cope scale, coping mechanism, medical interns, medical internship, mental wellbeing, mixed methods research, qualitative and quantitative research, stress coping, transition to internship, workplace stress

## Abstract

Introduction

Understanding the coping strategies adopted by medical interns is essential for promoting well-being and improving the quality of medical education. A mixed methods study was conducted to assess and explore this in a medical college in Bangalore, India.

Methods

An explanatory mixed methods study design was employed with a quantitative phase followed by a qualitative phase. The Brief-COPE (Coping Orientation to Problems Experienced Inventory) questionnaire was used to assess the coping strategies among 106 medical interns, and an in-depth interview was conducted among 10 of them. Descriptive statistics were reported as mean (SD) for continuous variables and frequency (proportions) for categorical variables. An independent sample t-test was performed to compare the means between groups. A p-value of <0.05 was considered statistically significant. Transcripts were prepared within 48 hours and analyzed by thematic analysis through manual coding.

Results

Emotion‑focused coping score was 27.92 (6.30), problem‑focused coping score was 20.29 (4.67), and avoidant coping score was 14.99 (3.98). Acceptance (mean = 5.53, SD = 1.50) was the most commonly used coping strategy, followed by positive reframing, planning, self‑distraction, and active coping, while behavioral disengagement (mean = 3.88, SD = 1.71), denial (mean = 3.42, SD = 1.51), and substance use (mean = 2.48, SD = 1.18) were the least utilized. Qualitative themes evolved based on quantitative findings, i.e., problem-focused coping, emotion-focused coping, and avoidant coping. Emotion-focused coping was the most commonly used coping strategy. Subthemes were talking to family and friends, relaxation through distraction, acceptance and normalization of workload over time, managing through planning and completing, engaging in physical activity, and faith-based practices.

Conclusion

Problem-focused coping (quantitative findings) and emotion‑focused coping (qualitative findings) emerged as the most commonly adopted coping strategies among medical interns. Acceptance, a subtype of emotion‑focused coping, was the most frequently used strategy. Age and gender differences were observed, with older interns favoring acceptance and male interns more often employing active coping. These findings highlight the strong need for awareness programs and structured interventions that promote the mental well‑being of medical interns.

## Introduction

Stress is a state of worry or mental tension, which is caused by a difficult situation. It is a natural human response that prompts addressing challenges and threats [1]. Coping is defined as the thoughts and behaviors mobilized to manage internal and external stressful situations [2]. Four major categories of coping are: (i) problem-focused (addresses the problem causing the distress), (ii) emotion-focused (aiming to reduce the negative emotions which is associated with the problem), (iii) meaning-focused (cognitive strategies is used by the individual in-order to derive and manage the meaning of the situation, and (iv) social coping (the individual reduces stress by seeking emotional or instrumental support from their community) [2].

Medical health professionals experience mental health problems due to professional pressures, work-life imbalance, and resilience issues [3]. A significant number of medical students suffer from stress associated with academic demands, living in campus accommodation, excessive workload, change in medium of teaching from secondary school to medical college [4-6]. Coping strategies reported by medical students, Interns, and doctors include task-oriented approaches such as working through stress and other approaches such as talking with co-workers, taking a time out, using humor, exercise, quiet time, spending time with family, self-directed learning, and avoidance [7-9].

Previous studies conducted in similar institutional settings have reported perceived stressors and coping strategies among medical interns [9-13]. However, there are limited studies in the literature exploring coping strategies among medical interns using a mixed-method study. Thus, a mixed methods study was conducted to assess and explore the coping strategies used by medical interns in a medical college in India.

## Materials and methods

This was an explanatory mixed methods study design that employed a quantitative phase followed by a qualitative phase, conducted in a medical college hospital in Bangalore, Karnataka, India, over two months, February and March 2024. A mixed methods design was deliberately chosen to capture both the structured assessment of coping strategies and the deeper contextual meanings behind them. The quantitative phase, using the Brief‑COPE (Coping Orientation to Problems Experienced Inventory) questionnaire, provided systematic scores across problem‑focused, emotion‑focused, and avoidant domains. Building on these findings, the qualitative interview guide was derived directly from the quantitative results, which helped in exploring why certain strategies were adopted and how they were experienced by medical interns. This sequential approach ensured that numerical data did not stand alone but was enriched and explained by lived narratives, offering a holistic understanding of coping mechanisms during the transitional phase of internship. 

The study was approved by the Vydehi Institutional Ethics Committee (approval number: VIEC/2024/APP/05). Detailed information pertaining to the nature, objectives of the study, and test procedures was provided to the study participants, and written informed consent was obtained. Anonymity of the study participants was ensured by assigning participant ID; data were stored in password-protected folders accessible only to the research team.

Study population and sample size

Medical interns who gave consent for the study were included. For the quantitative phase of the study, assuming that 78.3% of the subjects in the population have the factor of interest, the study required a sample size of 96 for estimating the expected proportion with 5% absolute precision and 95% confidence [6]. Sample size was calculated using Open Epi [14]. The sample size was further inflated by 10% to take care of non-response, incomplete responses, and refusals. This gave a sample size of 106. For the qualitative phase, 10 medical interns were chosen for in-depth interviews. Complete enumeration was done in order to reach the sample size.

Data collection and study tool

Data was collected in two phases: quantitative phase and qualitative phase. In the quantitative phase, data were collected by the interview method, which consisted of a socio-demographic profile and the Brief-COPE questionnaire.

The Brief-COPE is a 28-item self-report questionnaire designed to assess coping strategies [15-17]. Each item was measured on a four-point Likert scale, further categorized into 14 coping strategies by combining two items (minimum and maximum score observed was 2,8). The three overarching coping strategies according to the Brief-COPE questionnaire were classified as Problem-Focused Coping, Emotion-Focused Coping, and Avoidant Coping. The minimum and maximum scores in each category were 8-32, 12-48, and 8-32, respectively. The problem-focused category had active coping, use of informational support, positive reframing, and planning components; the emotion-focused category had emotional support, venting, humour, acceptance, self-blame, and religion components; and the avoidant coping category had self-distraction, substance use, denial, and behavioral disengagement components.

In the qualitative phase, an in-depth interview guide was developed based on the findings of the quantitative phase by the research team and validated by subject experts to ensure content validity. The interview guide helped in understanding the stressful situation, commonly used coping strategies, and how they helped in alleviating the stress. Ten medical interns were chosen based on convenience sampling. Interviews were audio-recorded after receiving written informed consent from the participants. Interviews lasted for a duration of 15-20minutes.

Statistical analysis

Data was entered in Microsoft Excel (Microsoft Corporation, Redmond, Washington, United States) and analyzed using IBM SPSS Statistics for Windows, version 23 (IBM Corp., Armonk, New York, United States). Normality of data was assessed using the Shapiro-Wilk test. Descriptive statistics were reported as mean (SD) for continuous variables and frequency (proportions) for categorical variables. An independent sample t-test was performed to compare means between groups. A p-value of <0.05 was considered statistically significant. Transcripts were prepared within 48 hours and analyzed by thematic analysis through manual coding.

## Results

Quantitative results 

A total of 106 participants were included in the study. Most participants were aged 22-23 years (n = 60, 56.6%), and 43.3% (n=46) were above 23 years. Most of the participants were female (n = 83, 78.3%), while 23 (21.6%) were male. The study population was predominantly Indian (n = 103, 97.1%), with a small representation from other nationalities (United States (n = 2, 1.8%); Kenya (n = 1, 1%)). With respect to residence, 81 (76.4%) were non-residential participants (commuting from home), and 25 (23.5%) were residential participants (living in campus hostels) (Table 1).

**Table 1:** Profile of study participants (N=106)

Variable	Categories	Frequency (Percentage)
Age	22-23	60 (56.6)
	>23	46 (43.3)
Gender	Female	83 (78.3)
	Male	23 (21.6)
Nationality	Indian	103 (97.1)
	Kenya	1 (1)
	Uniited States	2 (1.8)
Residence	Non-residential participants	81 (76.4)
	Residential participants	25 (23.5)

In the Brief-COPE questionnaire, the mean problem-focused coping score was 20.29 (SD = 4.67), emotion-focused coping was 27.92 (SD = 6.30), and avoidant coping was 14.99 (SD = 3.98) (Table 2). The problem-focused category scores were comparatively slightly higher than the emotion-focused category scores. 

**Table 2:** Brief-COPE scores in the coping strategies (three subscales) of the study participants (N=106) Brief-COPE: Coping Orientation to Problems Experienced Inventory

Coping strategies	Mean	SD
Problem focused	20.29	4.67
Emotion focused	27.92	6.30
Avoidant coping	14.99	3.98

Under the 14 subscales of the Brief-COPE questionnaire, the minimum score was 2, the maximum score was 8 (2.00=I haven't been doing this at all, 2.01 to 4.00=A little bit, 4.01 to 6.00 =A medium amount, 6.01 to 8.00=I’ve been doing this a lot). Subscale scores (2-8) were calculated by summing two items. The most commonly used coping strategies was acceptance (mean = 5.53, SD = 1.50), positive reframing (mean = 5.35, SD = 1.48), planning (mean = 5.28, SD = 1.50), self-distraction (mean = 5.22, SD = 1.47) and active coping (mean = 5.02, SD = 1.65). The least used coping strategies were behavioural disengagement (mean = 3.88, SD = 1.71), denial (mean = 3.42, SD = 1.51), and substance use (mean = 2.48, SD = 1.18) (Table 3).

**Table 3:** Brief-COPE scores in the coping strategies (14 subscales) of the study participants (N=106) Subscale scores (2-8) were calculated by summing two items Brief-COPE: Coping Orientation to Problems Experienced Inventory

Coping strategies	Mean	SD
Active coping	5.02	1.65
Informational support	4.64	1.64
Positive reframing	5.35	1.48
Planning	5.28	1.50
Emotional support	4.69	1.70
Venting	4.35	1.61
Humour	4.84	1.80
Acceptance	5.53	1.50
Religion	4.25	1.79
Self-blame	4.26	1.72
Self-distraction	5.22	1.47
Denial	3.42	1.51
Substance use	2.48	1.18
Behavioural disengagement	3.88	1.71

A summary of the 14 coping strategies is given in Table 4.

**Table 4:** Distribution of coping strategies (Brief-COPE scores) according to age, gender, and residential status of the study participant (N=106) Values given as mean±SD Brief-COPE: Coping Orientation to Problems Experienced Inventory

Coping Strategies	Age		Sex		Residential Status	
	22-23 (n=60)	>23 (n=46)	Male (n=23)	Female (n=83)	Residential (n=25)	Non-residential (n= 81)
Active Coping	5.03±1.68	5.00±1.63	5.70±1.71	4.83±1.59	5.01±1.61	5.04±1.83
Informational support	4.62±1.77	4.67±1.47	4.52±1.64	4.67±1.65	4.54±1.51	4.96±2.03
Positive reframing	5.32±1.39	5.39±1.61	5.22±1.65	5.39±1.44	5.21±1.49	5.80±1.41
Planning	5.13±1.59	5.48±1.36	5.39±1.03	5.25±1.61	5.30±1.45	5.24±1.69
Emotional support	4.60±1.78	4.80±1.60	4.30±1.55	4.80±1.73	4.54±1.66	5.16±1.79
Venting	4.47±1.67	4.20±1.54	3.91±1.56	4.47±1.61	4.19±1.56	4.88±1.69
Humour	4.92±1.76	4.74±1.86	4.83±1.80	4.84±1.81	4.69±1.77	5.32±1.81
Acceptance	5.25±1.62	5.89±1.26	5.96±1.36	5.41±1.53	5.48±1.43	5.68±1.74
Religion	3.98±1.76	4.59±1.79	4.74±2.09	4.11±1.68	4.28±1.84	4.12±1.64
Self-blame	4.33±1.68	4.17±1.78	4.00±1.50	4.34±1.77	4.28±1.67	4.20±1.89
Self-distraction	5.13±1.67	5.33±1.17	5.04±1.36	5.27±1.50	5.06±1.38	5.72±1.67
Denial	3.30±1.46	3.57±1.58	3.30±1.55	3.45±1.51	3.40±1.46	3.48±1.71
Substance use	2.50±1.30	2.46±1.00	2.52±1.20	2.47±1.18	2.51±1.24	2.40±0.95
Behavioural disengagement	3.75±1.75	4.04±1.67	3.61±1.69	3.95±1.72	3.80±1.67	4.12±1.85

An independent samples t-test was performed to compare coping strategies across the variables (age, gender, and residence). A statistically significant difference was observed for acceptance strategies (mean difference of -0.641; p = 0.029) between the age groups, with participants aged >23 years (5.89 ±1.26) reporting higher scores than those aged 22-23 years (5.25 ±1.62). A significant gender difference was also observed for active coping strategies [mean difference of 0.864 (p = 0.026)] with male participants (5.70 ± 1.72) reporting higher scores than female participants (4.83±1.59). No statistically significant differences were found for the other coping strategies across age, gender, or residence status (Table 5).

**Table 5:** Comparison of coping strategies with age, gender and residence

Coping scale	Age (22-23 years/'*≥*23 years)		Gender (Male/Female)		Residential Participants/Non-Residential Participants	
	Mean difference	P value	Mean difference	P value	Mean difference	P value
Active coping	0.033	0.919	0.864	0.026	-0.028	0.942
Informational support	-0.057	0.860	-0.153	0.695	-0.417	0.270
Positive reframing	-0.075	0.799	-0.168	0.634	-0.590	0.083
Planning	-0.345	0.244	0.138	0.698	0.056	0.871
Emotional support	-0.204	0.543	-0.491	0.223	-0.617	0.114
Venting	0.271	0.395	-0.557	0.144	-0.695	0.060
Humour	0.178	0.617	-0.017	0.968	-0.629	0.128
Acceptance	-0.641	0.029	0.547	0.124	-0.199	0.567
Religion	-0.604	0.086	0.631	0.136	0.164	0.691
Self-blame	0.159	0.638	-0.337	0.408	0.084	0.832
Self-distraction	-0.193	0.507	-0.222	0.526	-0.658	0.051
Denial	-0.265	0.375	-0.141	0.694	-0.085	0.808
Substance use	0.043	0.852	0.052	0.853	0.106	0.696
Behavioural disengagement	-0.293	0.386	-0.343	0.399	-0.318	0.421

Qualitative results 

Ten study participants were part of an in-depth interview. Among the 10 participants, 80% were female and 20% were male. The majority (90%) were of Indian nationality. In terms of age, 10% were 22 years old, 50% were 23 years old, and 40% were older than 23 years. Themes were evolved from a quantitative study, which included: Problem-Focused Coping, Emotion-Focused Coping, and Avoidant Coping, with subthemes of talking to family and friends, relaxation through distraction, acceptance and normalization of workload over time, managing through planning and completing, engaging in physical activity, and faith-based practices (Table 6).

**Table 6:** Summary of themes and subthemes

Theme	Subthemes	Verbatims
Emotion‑ Focused Coping	Talking to family and friends; acceptance and normalization of workload over time, faith-based practices	"I just call my parents or talk to my friends… it makes me feel lighter.” (P1). “After a while, you just accept that this is how it is.” (P4). "I listen to Islamic lectures; I do prayers and read Quran.” (P7)
Problem‑ Focused Coping	Managing through planning and completing	"I try to finish things early so I don’t get stressed later.” (P3).
Avoidant Coping	Relaxation through distraction, engaging in physical activity,	"I just watch something or sleep it off.” (P8). “Going to gym will help me cope with my stress.” (P6)

Talking to Family and Friends

The majority of the participants sought support through friends and family. Participants described talking to their loved ones, sharing their frustrations, seeking reassurance, and relying on trusted individuals to listen, validate, and calm them during stressful periods. Eight participants described venting out emotions to friends and family members helped in relieving stress.

“I just call my parents or talk to my friends… it makes me feel lighter.” 

“My hostel friends are the ones who keep me going.”

“I vent out to my friends, and then I feel better.”

Relaxation Through Distraction

The majority of the participants described using self-distraction as a coping strategy. Common activities included watching movies or series, listening to music, going out with friends, walking, shopping, or sleeping. These activities gave them a break and allowed them to “switch off” from clinical and academic pressures.

“I just watch something or sleep off.” 

“Music and going out help me forget everything for a while.”

Acceptance and Normalization of Workload Over Time

Several participants described gradually getting used to the demands of the internship. They observed that acceptance eased it for them to cope with stress.

“After a while, you just accept that this is how it is.” 

“As days passed by, I got the hang of it and it got better”.

Managing Through Planning and Completing

Only a few study participants engaged in activities such as planning, organising tasks, and finishing the tasks on time. These participants described that it helped in lowering the emotional overload.

 “I try to finish things early, so I don’t get stressed later.” 

“Trying to get everything done within campus and not carry things home”.

Engaging in Physical Activity

Two participants sought physical activity to alleviate stress by engaging in activities such as walking and going to the gym. They perceived it as an effective method and a proactive strategy to regulate their emotions and reduce stress.

“I will go out for a walk.”

“Going to the gym will help me cope with my stress.”

Faith-Based Practices

One participant reported that religious practices helped in coping with stress. Activities such as prayer, reading holy books, and watching spiritual lectures were perceived as sources of comfort. These practices provided hope and reduced anxiety when facing stressors.

*“I am a little spiritual person. If I go home, I listen to some Islamic lectures, I do prayers, and read the Quran. So I feel better while I am doing that.*”

## Discussion

This mixed methods study comprised a quantitative component (106 medical interns) and a qualitative component (10 medical interns). More than half of the study participants were in the age group of 22-23 years, the majority of them being female. A similar finding was observed in a study where the mean age of intern doctors was 24±2 years (the majority (46.6%) were 24 years of age), and female intern doctors were 51.66% [6].

Under the three subscales of the Brief-COPE questionnaire, the majority of study participants used problem-focused coping, followed by emotion-focused coping and avoidant coping. A study conducted in South India showed that emotion-focused coping was used by the majority of intern doctors [6], while another study reported task-oriented coping as the most common, followed by avoidance and emotion coping [7]. A study conducted in Malaysia among medical interns reported problem-focused coping as the main coping strategy, followed by emotion-focused and avoidance strategies [13]. Under the 14 subscales of the Brief-COPE questionnaire, the most commonly used coping strategy was acceptance, followed by positive reframing, planning, self-distraction, and active coping. Similar findings were reported in a study conducted in Malaysia [13] and another study conducted in Ethiopia [18], covering both the quantitative and qualitative findings. In the present study, the least used coping strategies were behavioural disengagement, denial, and substance use. Similar findings were reported in other studies [13,18]. While one study reported that 18% of medical interns relied on maladaptive coping strategies [6], another study reported 21% specifically involving substance use [11]. Variations in the findings while comparing the present study and existing literature could be due to regional variation, study methodology, and the nature of the stressful situation. Thus, the present study identified that acceptance coping (emotion-focused) was the most common coping strategy used by the study participants, followed by positive reframing, planning, active coping (problem-focused), self-distraction (avoidant coping), and the least being behavioural disengagement, denial, and substance use (Avoidant coping).

Study participants aged >23 years used acceptance coping strategies more than those aged 22-23 years in the present study, while a study among medical interns reported that younger interns were significantly more predisposed to psychological distress [13]. As students transition to professional roles, there is a change in the environment, the acceptance of fulfilling responsibilities, meeting the expectations of their roles, understanding and applying theoretical knowledge into practice, and handling stressful situations. Accepting these stressors would sometimes be challenging for the younger age group.

Male participants used active coping strategies more than females. While positive reappraisal was a significant coping strategy in both genders, followed by self-controlling, escape avoidance, and seeking social support, accepting responsibility was not used as a predominant coping strategy among both genders [12]. Gender disparities were highlighted in a scoping review of mental health challenges among doctors in India, as it provided insights on challenges faced by female doctors while balancing family responsibilities with long working hours and coping with societal expectations [3].

Within the qualitative component of the study, three themes and six subthemes were identified. The majority of the study participants felt talking to family and friends, and relaxation through distraction were coping strategies during stressful situations. Similar findings were reported in two studies: one highlighted coping strategies identified through interviews, such as working through stress, talking with co‑workers, taking a time out, using humor, and after‑work approaches like exercise, quiet time, and spending time with family [8]; another study found that the most commonly reported coping strategies were social support from friends, family, or colleagues and taking breaks or naps [11]. In the quantitative component, emotional support, coping, and self-distraction had higher scores, which are similar to the above qualitative findings.

In the present study, several participants accepted the situation over a period of time. Similar findings were reported in other studies [13,18]. Additionally, this finding is similar to the quantitative component of the study, i.e., acceptance coping of the emotion-focused component of the scale being the most common coping strategy used by the study participants. Thus, this study further emphasizes the importance of emotion-focused coping strategies.

Fewer participants relied on planning and organizing tasks, physical activity, and following religious rituals as a way of coping with stress. Studies reported task-oriented, working through stress, religious practices, and exercise as coping strategies [7,8,18]. In the quantitative component, study participants had a higher score for planning coping than for religious coping.

On summarizing qualitative findings, the emotion‑focused coping theme had a greater number of subthemes compared to the other domains, and more participants reported engaging in these strategies. This suggests that emotional regulation plays a particularly prominent role in how interns manage stress.

Strengths and limitations

Strength

A mixed methods study was conducted, and the findings of the quantitative component have been further validated by the qualitative component.

Limitation

As the study was conducted in one institute, the findings have limited generalizability; a multisite study would have strengthened the external validity. Selection and recall bias, as this is a cross-sectional study.

Implications

Medical interns employ a spectrum of coping strategies. These findings highlight the need for structured support systems, mentorship, and coping skills training during an internship to enhance resilience. The study contributes to the limited literature in this area and provides a foundation for future research and educational interventions

Recommendations

There is a strong need to develop awareness programs on coping strategies for medical interns during the orientation programme. With a focus on adopting coping strategies that promote mental well-being, there also needs to be follow-up sessions to reinforce the activity. Future research on a longitudinal study to compare and explore coping strategies adopted at various levels of the training period.

## Conclusions

Medical interns employ a spectrum of coping strategies. Problem-focused coping (quantitative findings) and emotion‑focused coping (qualitative findings) emerged as the most commonly adopted coping strategies among medical interns. Acceptance coping, a type of emotional coping, emerged as the most commonly used coping strategy, followed by positive reframing, planning, self-distraction, active coping, and the least used being behavioural disengagement, denial, and substance use. The older age group of study participants used acceptance coping strategies more than the younger one. Male participants used active coping strategies more than females. These findings highlight the need for structured support systems, mentorship, and coping skills training during an internship to enhance resilience. The study contributes to the limited literature in this area and provides a foundation for future research and educational interventions
